# Onsite training of doctors, midwives and nurses in obstetric emergencies, Zimbabwe

**DOI:** 10.2471/BLT.14.145532

**Published:** 2015-03-30

**Authors:** Joanna F Crofts, Teclar Mukuli, Bobb T Murove, Solwayo Ngwenya, Sma Mhlanga, Meluleki Dube, Elton Sengurayi, Cathy Winter, Sharon Jordan, Sonia Barnfield, Heather Wilcox, Abi Merriel, Sabelo Ndlovu, Zedekiah Sibanda, Sikangezile Moyo, Wedu Ndebele, Tim J Draycott, Thabani Sibanda

**Affiliations:** a School of Social and Community Medicine, University of Bristol, Bristol, BS10 5NB, England.; b Mpilo Central Hospital, Bulawayo, Zimbabwe.; c School of Clinical & Experimental Medicine, Birmingham Women's Hospital Foundation Trust, Birmingham, England.; d Morriston Hospital, Swansea, Wales.; e Royal Glamorgan Hospital, Cardiff, Wales.; f Bay of Plenty District Health Board, Whakatane Hospital, Whakatane, New Zealand.

## Abstract

**Problem:**

In Zimbabwe, many health facilities are not able to manage serious obstetric complications. Staff most commonly identified inadequate training as the greatest barrier to preventing avoidable maternal deaths.

**Approach:**

We established an onsite obstetric emergencies training programme for maternity staff in the Mpilo Central Hospital. We trained 12 local staff to become trainers and provided them with the equipment and resources needed for the course. The trainers held one-day courses for 299 staff at the hospital.

**Local setting:**

Maternal mortality in Zimbabwe has increased from 555 to 960 per 100 000 pregnant women from 2006 to 2011 and 47% of the deaths are believed to be avoidable. Most obstetric emergencies trainings are held off-site, away from the clinical area, for a limited number of staff.

**Relevant changes:**

Following an in-hospital train-the-trainers course, 90% (138/153) of maternity staff were trained locally within the first year, with 299 hospital staff trained to date. Local system changes included: the introduction of a labour ward board, emergency boxes, colour-coded early warning observation charts and a maternity dashboard. In this hospital, these changes have been associated with a 34% reduction in hospital maternal mortality from 67 maternal deaths per 9078 births (0.74%) in 2011 compared with 48 maternal deaths per 9884 births (0.49%) in 2014.

**Lessons learnt:**

Introducing obstetric emergencies training and tools was feasible onsite, improved clinical practice, was sustained by local staff and associated with improved clinical outcomes. Further work to study the implementation and effect of this intervention at scale is required.

## Résumé

**Problème:**

Au Zimbabwe, de nombreux établissements de santé ne sont pas en mesure de gérer les complications obstétricales graves. Selon les personnels de santé, c'est une formation inadaptée qui est le plus souvent identifiée comme le principal obstacle pour prévenir les décès maternels évitables.

**Approche:**

Nous avons conçu un programme local de formation aux urgences obstétricales, destiné au personnel de la maternité de l'Hôpital Central Mpilo. Nous avons formé 12 membres du personnel local pour assumer le rôle de formateurs et nous leur avons fourni tout le matériel et les ressources nécessaires pour organiser des formations. Ces formateurs ont dispensé des cours de formation d'une journée à 299 membres du personnel de l'hôpital.

**Environnement local:**

Au Zimbabwe, entre 2006 et 2011, la mortalité maternelle est passée de 555 à 960 décès pour 100 000 femmes enceintes. 47 % de ces décès sont considérés comme évitables. Pour la plupart, les cours de formation aux urgences obstétricales sont dispensés en externe, hors de l'établissement de santé, et ils ne concernent qu'un nombre limité de membres du personnel.

**Changements significatifs:**

Consécutivement au cours de formation des formateurs organisé au sein même de l'hôpital, 90 % du personnel de la maternité (138 personnes sur 153) a été formé localement, dès la première année, et à ce jour, 299 membres du personnel de l'hôpital ont été formés. Parmi les changements constatés localement, nous pouvons citer l'introduction de divers outils : tableau de planification pour les salles de travail, kits d'urgences, tableaux d'observation et d'alerte anticipée avec code couleur et tableau de bord de la maternité. Dans cet hôpital, ces changements ont permis une baisse de 34 % de la mortalité maternelle hospitalière : de 67 décès maternels pour 9 078 naissances (0,74 %) en 2011, l'hôpital est passé à 48 décès maternels pour 9 884 naissances (0,49 %) en 2014.

**Leçons tirées:**

L'introduction de cette formation aux urgences obstétricales et de divers outils a pu être mise en pratique sur le terrain, elle a permis d'améliorer les pratiques cliniques, elle a été soutenue par le personnel local et elle a conduit à une amélioration des résultats cliniques. Des travaux complémentaires doivent aujourd'hui être menés pour étudier la mise en œuvre et les effets de ce programme à d'autres échelles.

## Resumen

**Situacíon:**

En Zimbabwe, muchos centros de salud no pueden gestionar las complicaciones obstétricas graves. Según el personal, la formación inadecuada es el mayor obstáculo para la prevención de muertes maternas evitables.

**Enfoque:**

Se estableció un programa de formación en emergencias obstétricas in situ para personal de maternidad en el Hospital Central de Mpilo. Se formó a 12 miembros del personal local para convertirlos en instructores y se les proporcionó el equipo y los recursos necesarios para el curso. Los instructores realizaron cursos de un día para 299 miembros del personal del hospital.

**Marco regional:**

La mortalidad materna en Zimbabwe ha aumentado de 555 a 960 por 100.000 mujeres embarazadas de 2006 a 2011 y se cree que el 47% de las muertes son evitables. La mayoría de formaciones en emergencias obstétricas tienen lugar fuera del emplazamiento, lejos de la zona clínica, para un número limitado de miembros del personal.

**Cambios importantes:**

Tras un curso para formar a los instructores realizado en el hospital, se formó localmente al 90% (138/153) del personal de maternidad durante el primer año, con 299 trabajadores del hospital formados hasta la fecha. Los cambios en el sistema local incluyeron: la introducción de una cama en la sala de partos, cajas de emergencia, historiales de observación de alerta temprana con códigos de color y un panel de maternidad. En este hospital, estos cambios se han relacionado con una reducción del 34% en la mortalidad materna en el hospital, de 67 muertes maternas por 9.078 nacimientos (0,74%) en 2011 se pasó a 48 muertes maternas por 9.884 nacimientos (0,49%) en 2014.

**Lecciones aprendidas:**

La introducción de herramientas y formación en emergencias obstétricas fue posible in situ, mejoró la práctica clínica, recibió el apoyo del personal local y se relacionó con resultados clínicos mejorados. Se requiere más trabajo para estudiar la implementación y el efecto de esta intervención a escala.

## ملخص

**المشكلة:**

يتعذر على العديد من المرافق الصحيّة في زمبابوي إدارة المضاعفات الخطيرة للولادة. لقد أوضح العاملون في أغلب الحالات أن قلة التدريب هي العائق الأكبر في سبيل الحيلولة دون حدوث وفيات الأمهات التي يمكن تلافيها.

**الأسلوب:**

لقد قمنا بإنشاء برنامج تدريب في الموقع للحالات التوليدية الطارئة للعاملين في قسم التوليد بمستشفى Mpilo المركزي. قمنا بتدريب 12 عاملاً محلياً ليصبحوا مدرِبين/مدرِبات، ووفرنا لهم المعدات والموارد اللازمة للدورة. لقد عقد المدربون دورات لمدة يوم واحد لعدد 299 عاملاً في المستشفى.

**المواقع المحلية:**

لقد ****زاد معدل وفيات الأمهات في زمبابوي من 555 إلى 960 حالة وفاة لكل 100000 من السيدات الحوامل ما بين أعوام 2006 إلى 2011، ويُعتقد أن 47٪ من الوفيات كان يمكن تلافيها. يتم عقد معظم تدريبات الحالات التوليدية الطارئة خارج الموقع، بعيدًا عن المنطقة السريرية، لعدد محدود من العاملين.

**التغيّرات ذات الصلة:**

بعد دورة لتدريب المدرٍبين في داخل المستشفى، تم تدريب 90% (138/153) من العاملين في قسم التوليد محليًا خلال العام الأول، بمعدل 299 عاملاً مدرباً في المستشفى حتى الآن. تتضمن تغيّرات النظام المحلية: إنشاء مجلس لجناح التوليد، وعبوات للطوارئ، ومخططات الملاحظة للإنذار المبكر ذات ترميز بالألوان، ولوحة متابعة لحالات الولادة. في هذا المستشفى، ارتبطت هذه التغيرات بانخفاض بنسبة 34٪ في معدل وفيات الأمهات في المستشفى، من 67 حالة وفاة للأمهات لكل 9078 ولادة (0.74٪) في عام 2011 مقارنة مع 48 حالة وفاة للأمهات لكل 9884 ولادة (0.49٪) في عام 2014.

**الدروس المستفادة:**

كان تقديم الأدوات والتدريب على الحالات التوليدية الطارئة ممكنًا في الموقع، وقد أدى لتحسن الممارسة السريرية، وأصبح مستداماً من جانب العاملين المحليين، وارتبط بتحسن الحصائل السريرية. يُتطلب إجراء المزيد من العمل لدراسة تنفيذ وتأثير هذا التدخل على نطاق واسع.

## 摘要

**问题:**

津巴布韦境内许多医疗机构都无法处理严重的产科并发症。医务人员普遍认为，在预防可避免孕产妇死亡方面，缺乏足够的培训是最大的障碍。

**方法:**

我们在孟匹罗中心医院 (Mpilo Central Hospital) 为产科医务人员设立了产科急诊现场培训项目。我们将 12 名当地的医务人员培训为培训师，并向他们提供授课所需的设备和资源。培训师在该医院为 299 名医务人员举办了为期一天的课程培训。

**当地状况:**

从 2006 年到 2011 年，津巴布韦的孕产妇死亡率已从每 10 万名孕妇中 555 例上升到每 10 万名孕妇中 960 例，其中 47% 的死亡率被认为是可以避免的。多数产科急诊培训并非在现场进行，远离临床区域，且接受培训的医务人员数量有限。

**相关变化:**

在医院的培训师培训课程之后，90% (138/153) 的产科医务人员在第一年内就地接受了培训，至今已培训了 299 名医务人员。当地系统发生了以下改变：引进产房公告板、急救箱、早期预警观察色码图和产科仪表盘。在此医院中，随着这些改变的推行，已从 2011 年的每 9078 例分娩中发生 67 例孕产妇死亡降低至 2014 年的每 9884 例分娩中发生 48 例孕产妇死亡，孕产妇死亡率降低了 34%。

**经验教训:**

在现场引进产科急诊培训和工具是可行的；改善了临床实践；由当地医务人员持续进行，并且与改善的临床结果相关。在大范围内进一步研究这种干预措施的实施和效果是必需的。

## Резюме

**Проблема:**

В Зимбабве многие медицинские учреждения не могут справиться с серьезными акушерскими осложнениями. В большинстве случаев препятствием для предотвращения устранимой материнской смертности персонал называл недостаточную подготовку.

**Подход:**

В центральной больнице Мпило была организована программа подготовки по оказанию неотложной акушерской помощи для сотрудников родильного отделения, проводимая непосредственно на территории учреждения. В рамках программы было обучено 12 местных сотрудников, которые в результате сами получили квалификацию инструктора. Им было предоставлено оборудование и ресурсы, необходимые для самостоятельного проведения курса подготовки. Инструкторы провели однодневные курсы для 299 сотрудников больницы.

**Местные условия:**

Материнская смертность в Зимбабве возросла с 555 до 960 случаев на 100 000 беременных женщин в период с 2006 по 2011 г., при этом считается, что 47 % смертей можно было бы избежать. Большинство тренингов по оказанию неотложной акушерской помощи выездные. Они проводятся за пределами больниц для ограниченного числа сотрудников.

**Осуществленные перемены:**

После проведения тренинга для инструкторов в больнице в течение первого года 90 % персонала (138 из 153 сотрудников) родильного отделения прошли курс на местах. В настоящий момент этот курс обучения прошли 299 сотрудников. Изменения местной системы включали введение доски с информацией по родильной палате, аптечек для оказания неотложной помощи, цветных диаграмм наблюдения и раннего предупреждения, а также контрольных карт беременности. В рассматриваемой больнице изменения привели к уменьшению материнской смертности на 34 %: в 2011 г. наблюдалось 67 смертей на 9078 родов (0,74 %), а в 2014 г. количество смертей сократилось до 48 на 9884 (0,49 %).

**Выводы:**

Введение курсов обучения и предоставление инструментов для оказания неотложной акушерской помощи оказались осуществимыми на территории учреждения, положительно сказались на клинической практике, были поддержаны местными сотрудниками и привели к улучшению клинических исходов. Необходимы дальнейшие исследования реализации и последствий таких мероприятий в более широком масштабе.

## Introduction

Improving maternity care is a global priority, yet many health facilities in low-income countries are not able to manage obstetric complications adequately.^1^ Staff most commonly identified inadequate training as the greatest barrier to preventing avoidable maternal deaths. Training for obstetric emergencies may be part of the solution, but is not always effective: some training programmes either did not have a clinical effect or were associated with increases in perinatal morbidity. In low-income countries, some studies have reported an increase in knowledge or skills after training, but failed to demonstrate improved clinical outcomes,^2^^,^^3^ while other interventions failed to demonstrate improved skills.^4^

In high-income countries, effective obstetric emergency training is conducted within the clinical setting, involves a high proportion of relevant staff and implements practice-based tools such as emergency equipment boxes.^5^

In Zimbabwe, a programme of three-day off-site obstetric emergencies training was introduced in 2006.^6^ These courses were mainly attended by senior staff, with junior midwives and doctors, who provide most clinical care, unable to attend due to lack of alternative clinical cover. Staff considered that this training had not significantly improved clinical care. In 2011 a local maternal mortality review meeting at Mpilo Central Hospital recommended that all maternity staff should receive obstetric emergencies training.

## Local setting

Maternal mortality in Zimbabwe has increased from 555 to 960 per 100 000 live births from 2006 to 2011 and a national review of maternal deaths deemed that 47% of maternal deaths were avoidable.^7^

Mpilo Central Hospital is a public, tertiary referral hospital in Zimbabwe’s second largest city, Bulawayo. Mpilo Central Hospital manages approximately 10 000 births per year and provides all of the services defined by the World Health Organization’s (WHO’s) comprehensive emergency obstetric care services. These include administering antibiotics, uterotonic drugs (oxytocin) and anticonvulsants (magnesium sulfate); manual removal of the placenta, removal of retained products following miscarriage or abortion, assisted vaginal delivery, caesarean sections, safe blood transfusion, basic neonatal resuscitation and provision of care to sick and low-birth-weight newborns.^8^

## Multiprofessional training

Practical Obstetric Multi-Professional Training (PROMPT; http://www.promptmaternity.org) is a programme developed by midwives, obstetricians and anaesthetists that comprises: (i) an evidence-based skills development and training course; (ii) teamwork training incorporated in the clinical training; and (iii) a collection of tested local tools, checklists and local standardization techniques.

PROMPT emphasizes clinical practice over theory, with simulation in local clinical settings using practice based tools to help with correct decision-making during emergencies.

The implementation of PROMPT in the United Kingdom of Great Britain and Northern Ireland was reported to have improved knowledge,^9^ teamwork^10^ and clinical management in both simulation^10^ and clinical practice.^11^^,^^12^ Following the introduction of training, outcomes improved. Neonatal shoulder dystocia was reduced from 30/324 deliveries (9.3%) to 6/262 (2.3%) and hypoxic ischaemic encephalopathy decreased from 27.3 to 13.6 per 10 000 births.^11^ Improved outcomes after training were plausible, consistent and sustained.^13^ PROMPT has also been associated with improved perinatal outcomes in pilot sites in Australia^14^ and the United States of America.^15^

In November 2011, PROMPT training was introduced to Mpilo Central Hospital: First, a three-day train-the-trainers programme was held. The training was given by a team of seven staff from the United Kingdom (two midwives, three obstetricians, a paediatrician and an anaesthetist), three of whom were Zimbabwean. Twelve local staff (two midwifery matrons, four labour-ward midwives, two consultant obstetricians, two middle-grade obstetricians, a consultant paediatrician and a consultant anaesthetist) attended the programme to become trainers.

On day one, the team ran a demonstration of the PROMPT course. On day two, the 12 local staff were trained to deliver a local PROMPT course; working through lectures, running simulations on the labour ward and discussing the practicalities of local administration and implementation. On day three the local trained staff delivered an in-hospital PROMPT course attended by 15 other local staff. Local staff trainers and participants did not receive per diem payments.

The trainers were provided with the equipment and resources required to run training within their institution including trainer’s manuals, course manuals, and a digital versatile disc containing annotated presentations, videos, course timetables, evaluation sheets and certificates, together with two mannequins. One mannequin was used to teach the management of shoulder dystocia and vaginal breech birth (PROMPT birth trainer, Limbs and Things Ltd, Bristol, United Kingdom), and another to teach neonatal resuscitation - NeoNatalie (Laerdal Ltd, Stavanger, Norway).

## Outcome of PROMPT

Before the PROMPT course, only 31 (20%) staff of the 153 maternity staff at Mpilo Central Hospital had been to any relevant training; 26 of them had received their training off-site and just 11 staff had received any obstetric emergency training in the preceding 12 months.

PROMPT trained between 18 and 28 staff per one-day training course, with a total of 299 hospital staff trained. Within the first 12 months of training, 138/153 (90%) of staff who worked within the maternity unit had attended a PROMPT course. Midwives, midwifery tutors, nurses, laboratory technicians, pharmacists, junior doctors, obstetricians, paediatricians, anaesthetists and family doctors attended the training.

PROMPT appears to have empowered the staff to make local changes; improving self-reported teamwork and local interprofessional culture. Staff reported more confidence in their management of emergencies and a perception that emergency management has improved overall.

Following the train-the-trainers course, staff developed their own emergency boxes for the management of eclampsia and postpartum haemorrhage, based on those they used during simulated emergencies. Each box contains the equipment required to manage the first 10 minutes of the emergency with evidence-based management protocols. Boxes are kept at the nurse’s station and have been integrated into practice; their use is discussed at staff handovers and a member of staff is assigned to ensure the box is restocked after use. Maternity staff designed and implemented their own labour board containing information on labour progress, risk factors, actions required, and the staff member responsible for care, enabling staff to have an overview of all the patients on the labour ward.

To identify women at risk of complications, colour-coded early warning score charts were introduced. After the score charts were introduced, appropriate action in response to abnormal observations (e.g. starting antihypertensives or antibiotics) had increased from 1/24 (4%) to 11/15 (73%).^16^

## Clinical outcomes

Local monitoring of clinical outcomes is a key part of improving care quality and assessing the effect of training initiatives. In parallel with the implementation of PROMPT training, a monthly maternity dashboard of key clinical indicators has been introduced. Details of the maternity dashboard have been published.^17^ PROMPT training has been associated with a 34% reduction in maternal mortality at Mpilo Central Hospital from 67 maternal deaths per 9078 live births (0.74%) in 2011 compared with 48 maternal deaths per 9884 live births (0.49%) in 2014.

## Dissemination of training

Staff developed significant expertise in the adaptation of PROMPT training and tools to the Zimbabwean setting. Presentations of their experiences led to a demand for PROMPT training from other health facilities in Zimbabwe. To meet this demand, PROMPT training is being done in other facilities by volunteer staff from Mpilo Central Hospital with the support of the PROMPT Maternity Foundation. This work will help to define whether similar improvements can be implemented in smaller, rural health centres.

## Challenges and solutions

Implementing onsite obstetric emergencies training is challenging, especially in low-resource settings. Releasing staff from clinical work to attend or facilitate training can be difficult. Some staff expected additional payment to attend training, as most off-site training courses provide this.^18^ The involvement of Zimbabwean expatriates from the United Kingdom and New Zealand, who had a detailed understanding of health care training in both Zimbabwe and high-resource settings, was crucial in mitigating many issues encountered during the implementation of this training programme. Executive level support was also essential: the hospital executive mandated the participation of all maternity staff and this ensured that staff were released to attend training.

To maintain a sufficient faculty of trainers, six more midwives who demonstrated skill and enthusiasm during their attendance at a PROMPT course have been recruited as trainers by the local team.

## Costs

The total cost of providing training materials was 6000 United States dollars. The three most expensive items were a PROMPT birth trainer, a NeoNatalie and a laptop computer. This equipment has formed the basis of the hospital’s maternity training department and is also used for other training initiatives.

Once training has been established, the on-going costs of training are low. Training staff on-site eliminates travel, accommodation and hotel venue hire costs. A policy of no per diem payments has also reduced the cost of training. In addition, it may help to reverse a culture where training has become an opportunity to supplement income, rather than an opportunity to develop and disseminate professional skills.^18^

## Conclusion

PROMPT training has acted as a driver for quality improvement; improved the knowledge and skills of staff; self-reported team working and interprofessional culture and provided a place for staff to improve local systems (Box 1). The continuous monitoring and local reporting of clinical outcomes has also reinforced positive changes. Including local outcome data in the training helped to reframe the deaths of mothers and babies as everyone’s problem.

Box 1 **Summary of main lessons learnt**

- In low-resource settings, onsite training for obstetric emergencies is feasible.
- Practical training with quality improvement tools improves clinical practice and can be sustained by local staff.
- Success of training depends upon release of staff from clinical duties, use of practical simulation exercises in the local setting, leadership by experienced health professionals and monitoring of local outcomes to stimulate improvement.

It is unlikely that the training alone was responsible for the observed improvements in clinical outcomes. The success of the intervention is more likely to be a combination of factors, with the training being used as vehicle to introduce an improved professional culture and to trial and revise quality improvement tools. The use of these tools in clinical practice makes it easier for practitioners to do the right thing and this is a key factor in improving outcomes.

Training for obstetric emergencies is feasible in a low-resource setting. It empowers staff, improves local culture and can be sustained by local staff. Further work to study the implementation and effect of such interventions at scale and in different contexts is required.
